# EFFECT OF *PENICILLIUM* SPECIES ON THE ANTIBIOTIC RESISTANCE PROFILE OF *ALCALIGENES FAECALIS*

**DOI:** 10.21010/Ajidv18i2.2

**Published:** 2024-03-08

**Authors:** ALKHALIL S. Samia

**Affiliations:** Department of Clinical Laboratory Sciences, College of Applied Medical Sciences, Shaqra University, Alquwayiyah, Riyadh, Saudi Arabia

**Keywords:** *Alcaligenes faecalis*, Bacterial-fungal interaction, Antibiotic resistance

## Abstract

**Background:**

Infectious diseases due to antibiotic resistant pathogens are a global public health problem. This study aimed at determining the potential effect of bacterial–fungal interaction on the antibiotic susceptibility profile of *Alcaligenes faecalis.*

**Materials and Methods:**

*Alcaligenes faecalis* was isolated from water samples. The isolate was identified using the conventional biochemical tests and the 16S rRNA molecular sequencing technique. Additionally, *Penicillium* species was isolated and identified based on colony morphological characteristics and microscopic features. Standardized isolates were co-cultured in broth medium. Antibiotic susceptibility evaluation of the *Alcaligenes faecalis* from the co-culture and the original *Alcaligenes faecalis* was carried out using the Kirby bauer disk diffusion method.

**Results:**

The antibiotic susceptibility profile of *Alcaligenes faecalis* before and after co-culture remained largely unchanged except in the case of chloramphenicol, where the isolate showed reduced susceptibility. Molecular analysis of resistance gene revealed the absence of tested gene encoding antibiotic resistance, including the streptomycin resistance (str) genes (*stra* and *strb*) and the erythromycin resistance methylase (*erm*) gene.

**Conclusion:**

The result of this study showed that there is a minimal influence of *Penicillium* cultures on the susceptibility of *A. faecalis*. Further research involving a wide spectrum of microorganisms and their interactions should be conducted to acquire a thorough understanding of the influence of microbial interactions on antibiotic susceptibility profiles in order to pave way for novel strategies to combat antimicrobial resistance.

## Introduction

One of the major challenges of the twenty-first century has been the emergence and spread of antibiotic-resistant organisms, genes, and gene determinants (Ekwanzala *et al.*, 2018; Thabit *et al.*, 2023). Worldwide, antimicrobial resistance (AMR) is one of the top ten global public health challenges (Zhou *et al.*, 2022) and has been prioritized by the World Health Organization (Walsh *et al*, 2023).

Penicillin, erythromycin, fluoroquinolones, carbapenem, and antituberculosis medications are currently experiencing high rates of resistance in Saudi Arabia and other Middle Eastern nations (Zakai, 2019). According to the Institute for Health Metrics and Evaluation (2023), Saudi Arabia has the 14^th^ highest age-standardized mortality among 21 nations in the North Africa and Middle East regions with 2,500 fatalities attributable to AMR and 9,100 deaths related to AMR in Saudi Arabia in 2019.

*Alcaligenes faecalis* is a Gram-negative, non-fermenting obligate aerobe commonly found in soil, water and human intestinal flora. *Alcaligenes faecalis* is found in hospital settings where it has been isolated from stool, urine, wound discharge, blood, respiratory secretions and cerebrospinal fluid (Hasan *et al.*, 2019; Cantillo *et al.*, 2022) and a human opportunistic pathogen (Huang *et al.*, 2020).

Though not common *A. faecalis* has been implicated in various infections and diseases including bacteremia, endophthalmitis, meningitis, endocarditis, skin and soft tissue infections, urinary tract infections (UTIs), otitis media, peritonitis, and pneumonia (Brady and Leber, 2018; Huang *et al.*, 2020). The infection usually occurs in immunocompromised patients mostly in hospital environs through hospital devices and equipment or direct contact and fluids (Kavuncuoglu *et al.*, 2010; Hasan *et al.*, 2019).

*Alcaligenes faecalis* infection are often difficult to overcome due the increasing level of antibiotics resistance (Moscoso *et al.*, 2023). Various studies including Junejo *et al*. (2018), Hasan *et al*. (2019), Puah *et al*. (2019) and Huang *et al*. (2020) reported that this emerging pathogen have developed resistance of various broad-spectrum antimicrobial agents including penicillin, cephalosporins, nitrofurantoin, carbapenems, colistin trimethoprim/sulfamethoxazole, aminoglycosides, and quinolones. Puah *et al*. (2019) recorded an alarming increase of extended-spectrum β-lactamases in *A. faecalis*. Their study isolated the resistance gene *bla*TEM-116, and *bla*OXA-10 in *A. faecalis*. The *mcr-1*, *mcr-5* and *mcr-8* colistin resistance genes were reported in *A. faecalis* isolated from Humans, food and animals (chicken and pigs) by Anyanwu *et al*. (2021). Frequent use of antimicrobial agents in human and animals exposes various microorganisms including the microbiome consisting of trillions of organisms to the selective pressure of antibiotics (Schjørring and Krogfelt, 2011; Hoffmann *et al.*, 2016). Thus, these organisms become an important reservoir of antibiotic resistance (Bailey *et al.*, 2010; Sahoo *et al.*, 2012) and are released into the environment *via* excrements and other wastes (Kunhikannan *et al.*, 2021).

According to Nogueira *et al*. (2019) interactions between species of fungi and bacteria are complex, resulting in various forms of relationship such as antagonism, synergism, or neutral relationship. Hence, bacteria and fungi can interact in ways that either promote each other’s growth or result in competitive effects, potentially resulting in the inhibition of one and dominant growth of the other. Such interaction that is detrimental to at least one of the organisms in the association is termed Antibiosis, this usually occurs due to metabolites or cell components released (Ramírez Granillo *et al.*, 2015). Antibiosis often occurs due to self-defense, and struggle for limited resources and space (Moubasher *et al.*, 2022). Yu *et al*. (2017) stated that culturing of microbes in the same confined environment could stimulate the production of various secondary metabolites. Successful co-culturing experiments are archived *via* direct contact between the co-cultured organisms, bacterial cells and fungal mycelia interact and production of bioactive metabolites are studied (Knowles *et al.*, 2022).

Most fungal species are adaptive and easily respond to stress (Moubasher *et al.*, 2022). In most cases the antimicrobial substances produced by fungi are secondary metabolites not required for growth but mainly for self-defense and rivalry (Hani and Eman, 2015). Several studies on fungal–bacterial interactions demonstrated that co-culturing is a potential method for stimulating various secondary metabolites (Marmann *et al.*, 2014; Netzker *et al.*, 2015). Understanding antibiosis and antibiotic resistance is crucial for the development of effective strategies to combat the ever-increasing threat of antibiotic resistance. Hence, this study focuses on the potential effect of bacterial–fungal interaction in the development of alternative therapies to overcome antibiotic resistance.

## Materials and Methods

### Isolation and identification of *Alcaligenes faecalis*

One millimetre (1 ml) of water samples were serially diluted and 1 ml of 10^-2^ dilution of the water sample was dispensed into a petri dish, molten Eosin methylene blue (EMB) agar medium was dispensed into the petri dish and allowed to solidify before incubating at 37°C for 24 h and the plate was screened for the presence of discrete colonies of the target organism.

Characteristic distinct colonies of suspected *Alcaligenes* *faecalis* were repeatedly sub-cultured on fresh agar plates to obtain pure cultures which were stored on nutrient agar slant for further identification and analysis (Cheesbrough, 2006).

### Molecular identification of *Alcaligenes faecalis*

The 16s rDNA sequencing techniques was used to confirm the identity and characterize suspected *Alcaligenes* *faecalis* isolate using universal primers 27F and 1492R to amplify the 16S target region (Lane *et al.*, 1991; Turner *et al.*, 1999).

The genomic DNA extraction from the culture was performed using the Quick-DNA™ Fungal/Bacterial Miniprep Kit (Zymo Research, Catalogue No. D6005). Subsequently, the amplification of the 16S target region was carried out using the OneTaq® Quick-Load® 2X Master Mix (NEB, Catalogue No. M0486) and the primers outlined in Table 1. Gel electrophoresis was employed to assess the resulting products, which were then subjected to enzymatic clean-up utilizing the EXOSAP method. The sequencing of the extracted fragments in both forward and reverse directions was conducted using the Nimagen BrilliantDye™ Terminator Cycle Sequencing Kit V3.1, BRD3-100/1000. Purification of the sequenced fragments followed, utilizing the Zymo Research ZR-96 DNA Sequencing Clean-up Kit™, Catalogue No. D4050. Analysis of the purified fragments was performed using the ABI 3500xl Genetic Analyzer (Applied Biosystems, ThermoFisher Scientific) for each reaction across all samples. For sequence alignment, the BioEdit Sequence Alignment Editor version 7.2.5 was employed to analyze the .ab1 files generated by the ABI 3500XL Genetic Analyzer. The obtained results were further analysed via a BLAST search on NCBI for interpretation (Stephen et al., 1997).

**Table 1:** 16S amplification primers sequences

Primer	Target Sequence (5’ to 3’)
16S-27 F 16S rDNA sequence	AGAGTTTGATCMTGGCTCAG
16S-1492 R 16S rDNA sequence	CGGTTACCTTGTTACGACTT

### Isolation and identification of *Penicillium* sp

*Penicillium* species were isolated from milk samples. 1ml of the milk was added into a test tube containing 9mL of sterile water and a serial 4-fold dilution was made (Al-Mohanna, 2016). From the dilution, 0.5 mL of the suspension was inoculated on Potato dextrose agar and glass spreader was used to evenly spread the inoculum. The plates were incubated at 27°C ± 2°C. and observed every day for up to 5 days (Gaddeyya *et al.*, 2012).

Fungal isolate was identified based on colony morphological characterizations on the surface of the culture medium and microscopic features. The lactophenol cotton blue staining method outlined by Oyeleke and Manga (2008) was employed to identify the isolated fungi. Determination of Microscopic features was achieved by placing a drop of the lactophenol stain on a clean grease-free glass slide and a small portion of the aerial mycelia from the fungi culture was placed and spread in the drop of lactophenol stain and a cover slip is gently placed over it. The slide was viewed under a light microscope at ×10 and ×40 objectives.

### Fungal Bacterial Co-Culture

Isolated *Penicillium* sp. and *Alcaligenes faecalis* were cultured separately in Potato dextrose broth and Nutrient broth. The medium was incubated for 72 h at 27ºC ± 2ºC for fungi and 24 h at 37ºC ± 2ºC for the bacteria, after which each organism was standardized. Isolated *Penicillium* sp. and *Alcaligenes faecalis* were cultured separately on Potato dextrose agar and Nutrient broth. The media were incubated for 72 h at 27ºC ± 2ºC for fungi and 24 h at 37ºC ± 2ºC for the bacteria, after which each organism was standardized. The standardized bacteria contained 1.5x10^8^ cfu/ml, while the fungal culture was standardized to contain 1.0x10^6^ spore per ml. fungal–bacterial co-culture was performed by inoculating 0.5ml of the standardized isolates in Nutrient broth and incubating for 48 h. Fungal–bacterial coculture was performed by inoculating 0.5ml of the standardized isolates in Nutrient broth and incubating for 48 h.

### Subculture of *Alcaligenes faecalis* after Co-culture

Following a 2-day co-culture incubation period, *Alcaligenes* *faecalis* was sub-cultured from the co-culture broth onto MacConkey agar and subsequently incubated for 24 hs at 37°C.

### Antibiotic susceptibility test of *Alcaligenes faecalis*

Antibiotic susceptibility test of the *Alcaligenes faecalis* from the co-culture and a control *Alcaligenes faecalis* (original isolate) was carried out using the Kirby bauer disk diffusion method. Suspension of the test organism was prepared in normal saline to the turbidity of 0.5 McFarland standard and streaked onto Mueller Hinton agar (MHA). Antimicrobial discs of Trimethoprim-sulfamethoxazole (30μg), Gentamicin (10μg), Ciprofloxacin (10μg), Chloramphenicol (30μg), Reflacine (10μg), Streptomycin (10μg), Ofloxacin (10μg) and Amoxicillin (30μg) were aseptically applied onto the inoculated plates. The plates were left at room temperature (28ºC±2) for a few minutes before incubation for 18-24 h at 37°C. The inhibition zones around each disc was measured and interpreted in accordance with the breakpoints and criteria recommended by the Clinical Laboratory Standards Institute (CLSI, 2018) and the susceptibility profile of the two isolates were compared.

### Molecular identification of Antibiotic resistance genes in *Alcaligenes faecalis*

The Quick-DNA Fungal/Bacterial Miniprep Kit (Zymo Research, Catalogue No. D6005) was used to extract the genomic DNA extraction of the isolate. The procedure involved adding 50100mg of wet weight bacterial cells to a ZR BashingBead™ Lysis Tube (0.1 and 0.5 mm) along with 750μl of BashingBead™ Buffer. The tube was processed in a bead beater (Disruptor Genie) for 20 minutes, followed by centrifugation of the ZR BashingBead™ Lysis Tubes (0.1 and 0.5 mm) at 10000 x g for 1 minute. The resulting supernatant was filtered using a ZymoSpin™ III-F Filter, and Genomic Lysis Buffer was added to the filtrate. Further purification was performed using Zymo-Spin™ IICR Columns, with multiple wash steps. Finally, DNA was eluted using DNA Elution Buffer, resulting in the extraction of high-quality genomic DNA for analysis. The quality and quantity of the extracted DNA was measured using a nanodrop (Thermo Scientific™ NanoDrop™ One Microvolume UV-Vis Spectrophotometer). The target region was amplified using OneTaq® Quick-Load® 2X Master Mix (NEB, Catalogue No. M0486) following the conditions presented in Table 2. The primers used for the reaction are presented in Table 3.

**Table 2:** Target region amplification conditions

Component	Volumes for a 25μL reaction
Template DNA	2-4μL
10 μM Forward Primer	0.5μL (10nM)
10 μM Reverse Primer	0.5μL (10nM)
One Taq Quick Load 2X Master Mix with Standard Buffer	12.5μL
Nuclease free water	Varies (25-total of volumes above) μL

**Table 3:** Primers used for amplification

Name of Primer	Target Sequence
STRA	TATCTGCGATTGGACCCTCTG
STRB	CATTGCTCATCATTTGATCGGCT
ERMA F	ACGATATTCACGGTTTACCCACTTA
ERMA R	AACCAGAAAAACCCTAAAGACACG

The extracted deoxyribonucleic acid (DNA) samples underwent polymerase chain reaction (PCR) amplification using the Eppendorf Mastercycler nexus gradient 230 thermal cycler. The amplification process included an initial denaturation step at 94°C for 5 min, followed by a series of denaturation, annealing, and extension cycles. Denaturation steps were performed at 94°C for varying durations, while annealing occurred at temperatures ranging from 50°C to 55°C for 30 seconds each. Extension steps were carried out at 68°C for 1 minute and 30 seconds. The PCR process concluded with a final extension step at 68°C for 10 minutes.

After PCR amplification, 2 ul of each PCR product underwent gel electrophoresis on a 1% agarose gel, stained with SafeView Red, and visualized using a gel documentation system (EBOX, Vilber Lourmat, Italy).

The PCR products were subjected to a purification process using the ExoSAP enzymatic method. Sequencing of DNA fragments was carried out using the Nimagen Brilliant Dye™ Terminator Cycle Sequencing Kit V3.1 (BRD3-100/1000) following the manufacturer’s instructions. The labelled sequencing products were further purified using the ZR-96 DNA Sequencing Clean-up Kit (Catalogue No. D4053) before being analysed on the Applied Biosystems ABI 3500XL Genetic Analyzer with a 50cm array using POP7. The resulting sequence data were collected.

## Results

### Identity of test organisms

The morphological characteristics and biochemical tests show the bacterial isolate to be *Alcaligenes* sp (Table 4). Plate I show the culture of *Alcaligenes* sp. on EMB agar plate (Plate 1a) and the fungus *Penicillium* sp on PDA (Plate 1b). Macroscopic and microscopic analysis, ensures a reliable and accurate identification of fungal species, from the visual examination of fungal isolate on agar plates and microscopy (Plate 1b and 1c), observed features were consistent with the fungal species *Penicillium*. The isolate displayed distinctive characteristics such as its green colour, powdery texture and white edges on agar plates. Microscopic examination revealed unique structures particularly the presence of grouped phialides (brush like structure) at the apex.

**Table 4:** Biochemical characteristics of *Alcaligenes* sp.

Basic Characteristics	Properties
Gram Staining	- rod
Catalase	+
Citrate	+
Gelatin Hydrolysis	-
Indole	-
Motility	+
Nitrate Reduction	-
Oxidase	+
Spore	-
Urease	-

**Plate I:**
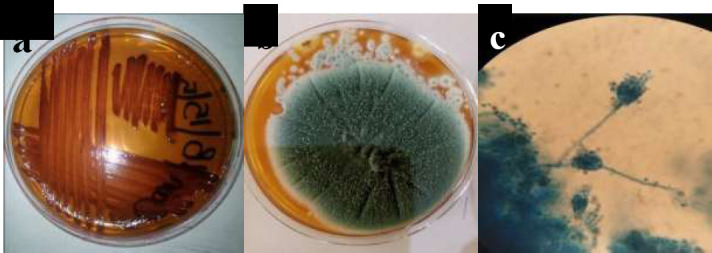
**(a)** *Alcaligenes* *faecalis* isolate on Eosin methylene blue (EMB) agar: Macroscopic **(b)** 254 and Microscopic **(c)** Features of *Penicillium* sp.

**Figure 1:**
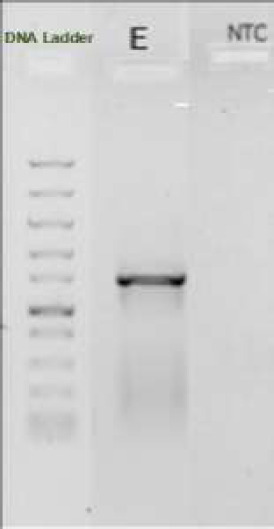
Agarose gel electrophoresis image that show the PCR product analysis of the 16S 259 rRNA target region of the test isolate (*Alcaligenes faecalis*) **E:** *Alcaligenes faecalis*, **NTC:** Non-template control. A reaction that contains all the PCR components,but nuclease-free water is used as a template instead of DNA. It is used as a quality control point.

**Figure 2:**
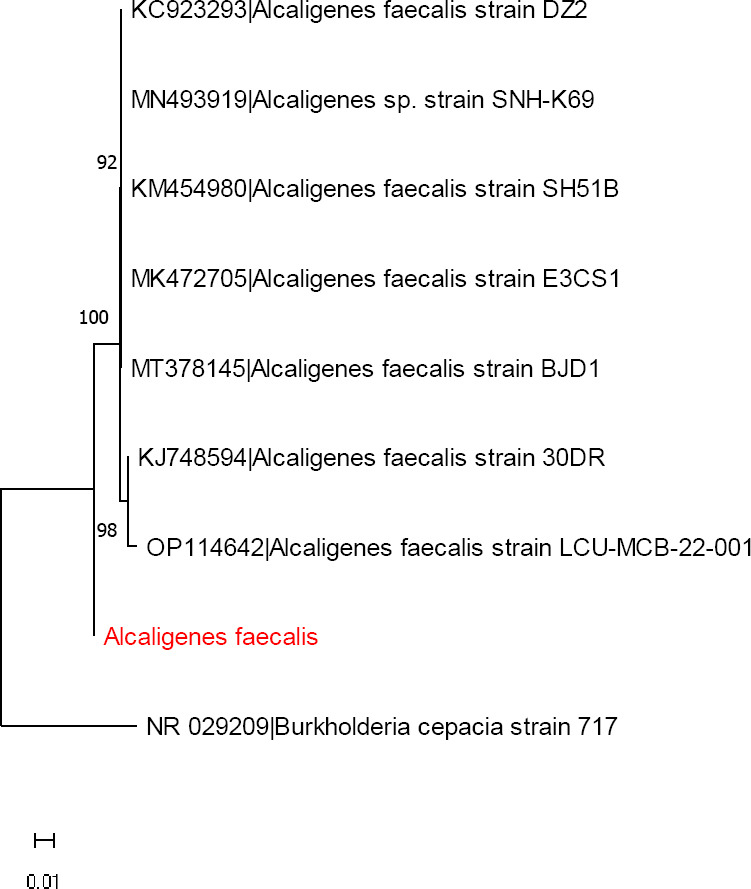
Phylogenetic Tree of *Alcaligenes* *faecalis* based on its 16S rRNA Gene Sequences

The identity of the bacterial isolate was confirmed using the 16S rRNA sequencing analysis. The BLAST results of the bacterial DNA sequence corresponded to the similarity between the sequence queried and the biological sequences within the NCBI database. The predicted organism was *Alcaligenes faecalis* with a percentage ID of 99.17% and accession number KF641853.1. The agarose gel electrograph indicating 16S rRNA target region amplification of the test isolate (*Alcaligenes faecalis*) is shown in Figure 1. The phylogenetic tree of the test isolate*, Alcaligenes faecalis*, constructed to elucidate its evolutionary relationships and origin and showing the relationship of the test isolate with other *Alcaligenes faecalis* and *Burkholderia cepacian* is presented in Figure 2.

### Antibiotic Susceptibility Zones of Inhibition (mm)

The antibiotic susceptibility test results of *Alcaligenes faecalis* isolate before and after coculturing is shown in Table 5. The susceptibility profile (mm) of the isolate before and after coculture (Plate II) remained largely unchanged except in the case of chloramphenicol, were the isolate showed reduced susceptibility.

**Table 5:** Antibiotic susceptibility test results of *Alcaligenes faecalis* isolate before and after coculture.

Sample code	CPX (mm)	CH (mm)	PEF (mm)	S (mm)	SXT (mm)	OFX (mm)	CN (mm)	AM (mm)
Before co-culture	37 (S)	11 (R)	29 (S)	- (R)	- (R)	30 (S)	11 (R)	- (R)
After co-culture	33 (S)	13 (I)	30 (S)	- (R)	- (R)	32 (S)	12 (R)	- (R)

Keys: CPX: Ciprofloxacin; CH: Chloramphenicol; PEF: Reflacine; S: Streptomycin; SXT: Trimethoprin-Sulfamethoxazole; OFX; Ofloxacin; CN: Gentamicin; AM: Amoxicillin

**Plate II:**
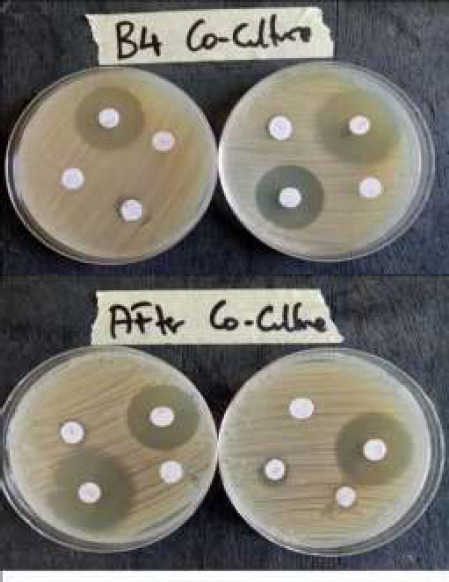
The susceptibility profile of *Alcaligenes faecalis* before and after coculturing with *Penicillium* sp.

### Identification of Antibiotic resistance genes in *Alcaligenes faecalis*s

**Figure 3:**
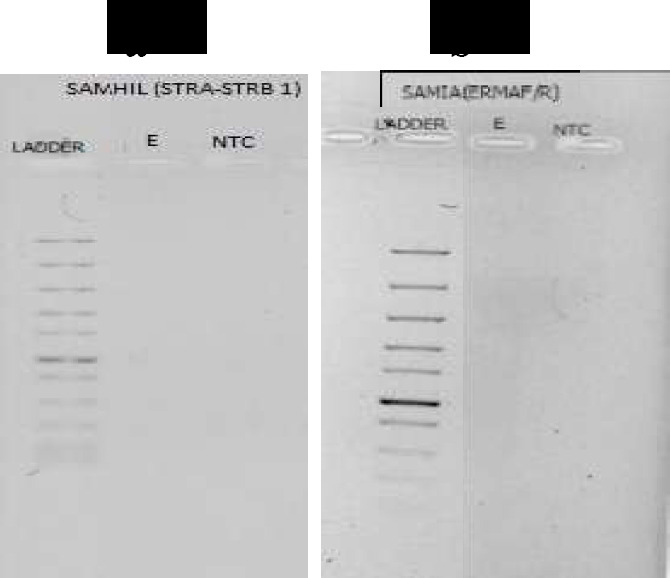
Agarose gel electrophoresis image that show the PCR product analysis of the test isolate showing negative amplification of the *stra* and *strb* target region **(a)** and negative amplification for erm target region **(b)**

Molecular analysis to detect resistance genes (*stra, strb*, and *erm*) in the *Alcaligenes* *faecalis* isolate yielded negative results, indicated by the absence of amplification for the target genes on the gel (Figure 3). Suggesting that the test isolate does not harbor detectable levels of the selected antibiotic resistance genes.

## Discussion

*Alcaligenes faecalis* is an emerging potential pathogen, that has *been* reported to show decreased susceptibility to commonly used antibiotics (Huang *et al.*, 2020). In the current study, the antibiotic susceptibility profile of the *A. faecalis* isolate before and after co-culture remained the same except for chloramphenicol. *A. faecalis* after co-culture showed reduced susceptibility to chloramphenicol. This result contradicts the finding of a study by Moubasher *et al*. (2022) on the impact of bacterial–fungal interaction on carbapenem-resistant *Klebsiella pneumoniae*. Their study reported that *K. pneumoniae* isolates that were previously resistant to carbapenems (meropenem and imipenem) exhibited a change in susceptibility after co-culture with the fungus *Scopulariopsis brevicaulis*. The difference in findings could be attributed to the variation in the characteristics and adaptability of microbial species involved in each study, nature of the interaction between the fungal and bacterial species and the mechanism of resistance utilized by each bacterium species (Frey-Klett *et al.*, 2011; Deveau *et al.*, 2018). In addition, difference in experimental conditions such as the duration and intensity of the co-culture can also influence the outcome of the study.

Antimicrobial resistance has been identified as one of the most pressing global problems (Prestinaci *et al.*, 2015). The antibiotic susceptibility profile of *Alcaligenes faecalis* isolates revealed resistance to commonly prescribed antibiotics Chloramphenicol, Streptomycin, Trimethoprin-Sulfamethoxazole, Gentamicin and Amoxicillin. These antibiotics are traditional first-line therapeutic drug options for treating Gram-negative bacterial infections (DelgadoValverde *et al.*, 2013; Otokunefor *et al*. 2018). Several other studies also reported resistance to various other antibiotics in *A. faecalis*. Puah *et al*. (2019) recorded resistance to cefazolin, trimethoprim/sulfamethoxazole, ampicillin/sulbactam, cefepime, tobramycin, ciprofloxacin, and nitrofurantoin. Moscoso *et al*. (2023) observed resistance to penicillin, cephalosporins, carbapenems, aminoglycosides, and quinolones. This level of resistance to first line drugs in nonclinical isolates poses a major public health concern. The observed level of resistance in non-clinical

*A. faecalis* could be attributed to intrinsic resistance mechanisms and acquisition of resistance genes (Lang *et al.*, 2022). Transmission and acquisition of resistance genes and other DNA sequences such as drug resistance plasmids via horizontal transfer plays a critical role in the development of bacterial resistance to antibiotics (Liu *et al.*, 2020). The environment has been reported to contribute to antimicrobial resistance. Larsson and Flach, (2022) indicated the environment as a source of antimicrobial resistance genes. Furthermore, Lykov and Volodkin, (2021) reported the aquatic ecosystems as an important reservoir of antibiotic resistant organisms and resistance genes due to pollution by human waste, agricultural and industrial wastewater. The observed increased resistance to aminoglycoside, streptomycin and gentamicin in this study is mostly attributed to the presence of the aminoglycoside resistance genes.

Aminoglycosides are important broad-spectrum antibiotics that inhibit protein synthesis via binding to the 16S rRNA (Asghar and Ahmed, 2018). Various intrinsic and acquired mechanisms are responsible for resistance to aminoglycosides. Several mechanisms contribute to the development of resistance to aminoglycoside, although the presence of aminoglycoside modifying enzymes encoded by various aminoglycoside resistance genes is the most clinically and epidemiologically significant (Soleimani *et al.*, 2014).

The current study observed significant resistance to streptomycin even though the streptomycin resistance (*str*) genes *stra* and *strb* were not detected in the *Alcaligenes* *faecalis* isolate. As such the molecular basis for the observed resistance in this study could be attributed to other aminoglycoside resistance genes or various intrinsic resistance mechanisms present in the isolate. Numerous other genes that can encode for streptomycin resistance has been documented in various studies. Resistance determinants such as the *aadA* and *ant* genes that encode enzymes that modify aminoglycosides including streptomycin by phosphorylation or adenylation thus reducing the streptomycin molecule’s binding affinity to the bacterial ribosome, thereby conferring resistance to the antibiotic have been reported by Prabhu *et al*. (2020). Lee *et al*. (2021) reported streptomycin resistance gene *strA*, *strB*, and *aadA* genes as the most widely distributed streptomycin resistance determinants. The gene *aph*(E) and *sat*(3) were reported by Ammor *et al*. (2008) to also encode streptomycin resistance. Intrinsic mechanisms including ribosomal mutations and modifications, cell membrane modification and efflux pumps have also been identified to cause streptomycin resistance (GarneauTsodikova and Labby, 2016; Dal Molin *et al.*, 2017).

According to Wachino and Arakawa, (2012) and Krause *et al*. (2016) aminoglycoside resistance could result from the modification of the aminoglycoside target site. Aminoglycoside inhibits protein synthesis via binding to the A-site on the 16S ribosomal RNA of the 30S ribosome (Hong *et al.*, 2014; Wilson *et al.*, 2014; Arenz and Wilson, 2016). Krause *et al*. (2016) pointed out that Methylation of specific rRNA nucleotide residues within the 16S rRNA prevents aminoglycosides from effectively binding to their target. This modification is enzymatically catalysed by the 16S rRNA methyltransferases (RMTases). These RMTases can be acquired by, and from other bacterial species, often via horizontal transfer of plasmid containing the RMTase gene (Garneau-Tsodikova and Labby, 2016). This mechanism of methylation mediated resistance significantly contributes to the reduced susceptibility to aminoglycoside antibiotics (Wang *et al.*, 2023). Likewise, the Erythromycin Resistance Methylase (*erm*) gene was not identified in the current study. It is important to note that while specific resistance genes like the *stra, strb* and *erm* may not have been detected, other resistance genes and diverse mechanisms could still potentially contribute to the observed reduced susceptibility.

Additionally, it is essential to acknowledge that inappropriate or suboptimal use of antibiotics is an important factor in resistance development. Selective pressure from the widespread use of antibiotics across clinical settings, agriculture, and the environment have been established as a driving force behind the development and spread of antibiotics resistance (Da Costa *et al.*, 2013; Gaze *et al.*, 2013; Ayukekbong *et al.*, 2017; Bengtsson-Palme *et al.*, 2018; Iwu *et al.*, 2020; Ahmad *et al.*, 2021; Mutuku *et al.*, 2022).

## Conclusion

Fungal-bacterial co-culture of *Alcaligenes faecalis* and *Penicillium* sp. had little to no impact on the antibiotic susceptibility profile of *Alcaligenes faecalis*. The antibiotic susceptibility profile of the isolate remained the same except for chloramphenicol where a reduced susceptibility was observed, indicating a potential development of resistance. Further research involving a wide spectrum of microorganisms and their interactions should be conducted to thoroughly understand the influence of microbial interactions on antibiotic susceptibility profiles to pave the way for novel strategies to combat antimicrobial resistance.

In addition, resistance observed in nonclinical *Alcaligenes faecalis* isolate to various commonly prescribed antibiotics reinforces the importance of an antimicrobial stewardship program and the urgent need for new therapeutic alternatives to successfully manage antimicrobial-resistant organisms.

### Conflict of interests:

The author declares that there are no competing interests associated with this study.

**List of Abbreviations:**
UTIs -: urinary tract infections
EMB -: Eosin methylene blue
MHA -: Mueller Hinton agar
DNA -: deoxyribonucleic acid
PCR -: polymerase chain reaction
