# EFFECT OF *PENICILLIUM* SPECIES ON THE ANTIBIOTIC RESISTANCE PROFILE OF *ALCALIGENES FAECALIS*

**DOI:** 10.21010/Ajidv18i2S.6

**Published:** 2024-07-04

**Authors:** ALKHALIL S. Samia

**Affiliations:** Department of Clinical Laboratory Sciences, College of Applied Medical Sciences, Shaqra University, Alquwayiyah, Riyadh, Saudi Arabia

In the version of this article initially published, there were errors resulting from merged author names.

The following sentences highlight the errors and the corrected details:

**Alkhalil S.S., Afr., J. Infect. Dis. (2024) 18 (2): 8-18**

https://doi.org/10.21010/Ajidv18i2.2

EFFECT OF *PENICILLIUM* SPECIES ON THE ANTIBIOTIC RESISTANCE PROFILE OF *ALCALIGENES FAECALIS*

ALKHALIL S. Samia*

Department of Clinical Laboratory Sciences, College of Applied Medical Sciences, Shaqra University, Alquwayiyah, Riyadh, Saudi Arabia

^*^Corresponding Author’s E-Mail: salkhalil@su.edu.sa

**Should read:**

**ALKHALIL S. Samia ., Afr., J. Infect. Dis. (2024) 18 (2S): 34**

https://doi.org/10.21010/Ajidv18i2S.6

EFFECT OF *PENICILLIUM* SPECIES ON THE ANTIBIOTIC RESISTANCE PROFILE OF *ALCALIGENES FAECALIS*

ALKHALIL S. Samia*

Department of Clinical Laboratory Sciences, College of Applied Medical Sciences, Shaqra University, Alquwayiyah, Riyadh, Saudi Arabia

^*^Corresponding Author’s E-Mail: salkhalil@su.edu.sa

